# Multimodality Imaging of Cardiac Myxomas

**DOI:** 10.31083/j.rcm2506204

**Published:** 2024-06-03

**Authors:** Maja Hrabak-Paar, Miroslav Muršić, Tihana Balaško-Josipović, Daniel Dilber, Nikola Bulj

**Affiliations:** ^1^Department of Diagnostic and Interventional Radiology, University Hospital Center Zagreb, 10000 Zagreb, Croatia; ^2^University of Zagreb School of Medicine, 10000 Zagreb, Croatia; ^3^Department of Pediatrics, University Hospital Center Zagreb, 10000 Zagreb, Croatia; ^4^Department of Cardiology, University Hospital Centre “Sestre Milosrdnice”, 10000 Zagreb, Croatia

**Keywords:** cardiac myxoma, cardiac masses, echocardiography, computed tomography, magnetic resonance imaging

## Abstract

Cardiac myxomas are the most common benign cardiac neoplasms. Echocardiography is the first-line imaging modality used to analyze cardiac masses, allowing the detection of tumor location, size, and mobility. However, additional imaging techniques are required to confirm the diagnosis, evaluate tissue characteristics of the mass, and assess potential invasion of surrounding structures. Second-line imaging includes cardiac magnetic resonance imaging (MRI) and/or computed tomography (CT) depending on availability and the patient’s characteristics and preferences. The advantages of CT include its wide availability and fast scanning, which allows good image quality even in patients who have difficulty cooperating. MRI has excellent soft-tissue resolution and is the gold standard technique for noninvasive tissue characterization. In some cases, evaluation of the tumor metabolism using 18F-fluorodeoxyglucose positron emission tomography with CT may be useful, mainly if the differential diagnosis includes primary or metastatic cardiac malignancies. A cardiac myxoma can be identified by its characteristic location within the atria, typically in the left atrium attached to the interatrial septum. The main differential diagnoses include physiological structures in the atria like crista terminalis in the right atrium and the coumadin ridge in the left atrium, intracardiac thrombi, as well as other benign and malignant cardiac tumors. In this review paper, we describe the characteristics of cardiac myxomas identified using multimodality imaging and provide tips on how to differentiate myxomas from other cardiac masses.

## 1. Introduction

Multimodality imaging is essential for cardiac mass assessment, enabling detection and characterization as well as evaluation of size, mobility, and invasion of surrounding structures. In symptomatic patients, cardiac masses are typically detected using echocardiography. They may also be detected incidentally during thoracic imaging—mainly computed tomography (CT) or magnetic resonance imaging (MRI) - in patients without symptoms related to the mass. Characterization of cardiac masses using imaging is based on location and tissue characteristics.

We aim to review imaging techniques used to evaluate cardiac masses (echocardiography, CT, and MRI) and the morphological characteristics of cardiac myxomas based on multimodality imaging. We will also review differential diagnoses and provide tips on making the correct diagnosis.

## 2. Echocardiography

**Fig. 1.**
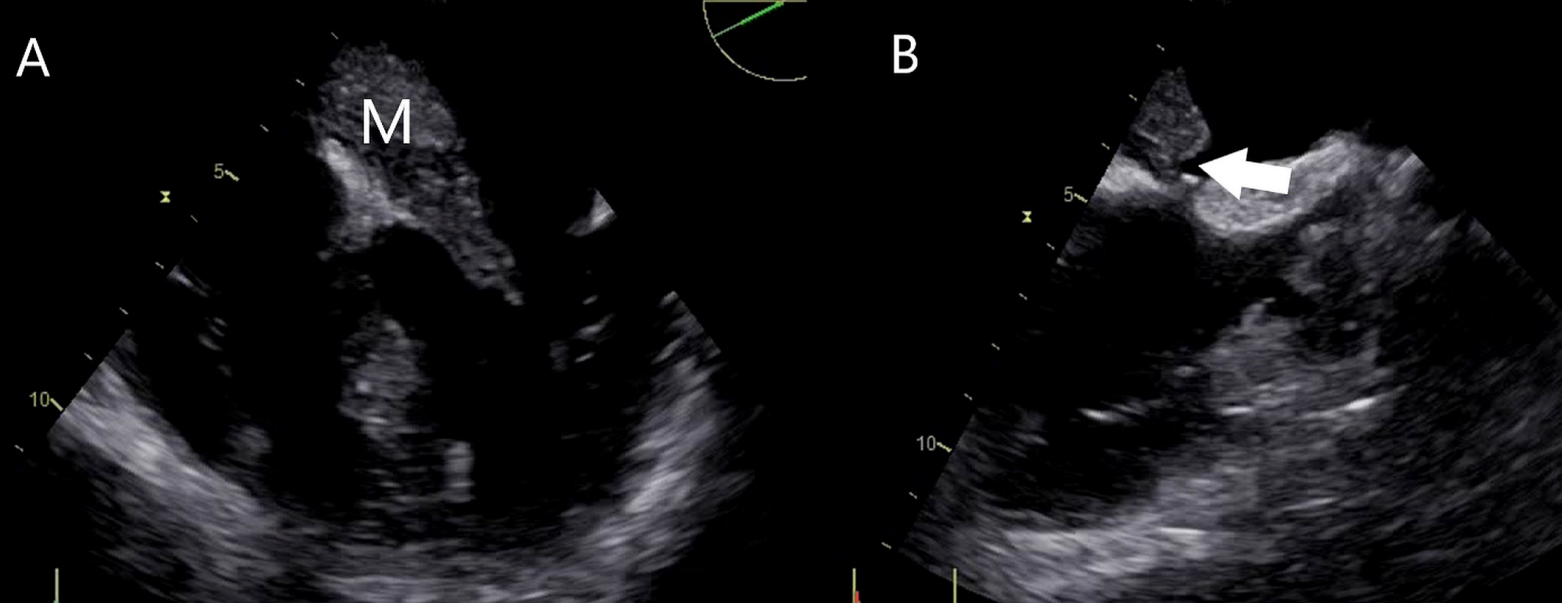
**Transesophageal echocardiography shows a large left atrial myxoma.** (A) The myxoma (M) protrudes into the left ventricle with partial mitral valve obstruction. (B) The mass is attached by a stalk (arrow) to the interatrial septum at the fossa ovalis.

Echocardiography is the primary imaging method for diagnosing cardiac myxomas [1, 2, 3] and many patients are identified during routine echocardiographic examination. This technique provides information on the size and localization of the tumor, as well as data on its shape, mobility, and relationships with surrounding structures. It also helps to rule out differential diagnoses such as thrombus or other cardiac tumors. During the procedure, the hemodynamic consequences of intracardiac tumor masses can be analyzed in detail, including dysfunction and obstruction of valvular flow, and obstruction of inflow to the heart (Fig. 1). In 75–80% of cases, echocardiography shows the myxoma as a mobile mass located in the left atrium. It grows from or is fused with the interatrial septum, most often at the fossa ovalis (typical localization). Approximately 15–20% of myxomas originate in the right atrium, while valvular or ventricular localization is very rare [4, 5]. Most tumors are attached to the underlying endocardium by a stalk.

Transthoracic echocardiography is very reliable for identifying larger tumors in the left atrium. However, transesophageal echocardiography increases the sensitivity and specificity of the examination, especially for smaller tumors and those in atypical locations like the right atrium [6, 7]. Transesophageal echocardiography with high-frequency transducers has improved spatial resolution compared with the transthoracic method. Transesophageal echocardiography (particularly three-dimensional imaging) is also superior for detecting the site of tumor contact with underlying heart tissue as well as the stalk. Tumors usually show different degrees of echogenicity with areas of calcification [8, 9, 10]. Echocardiographic contrast agents can help evaluate mass vascularization. Contrast hyperenhancement is often apparent in malignant tumors, whereas myxomas tend to be less or partially enhanced in contrast studies [11].

Echocardiographic examinations most often identify myxomas of two distinct morphological types. The first type includes polypoid myxomatous tumors, which are usually large and mobile with a smooth surface and are responsible for obstruction of valvular inflow, usually the mitral valve due to protrusion into the left ventricle. Areas with different echogenicity due to cystic regions and hemorrhage are seen in these tumors. The second type includes papillary myxomas, which are smaller than the polypoid type and are characterized by multiple villi. These myxomas are more likely to be associated with embolic events [12].

Echocardiographic examinations are useful for differentiating myxomas from thrombi. Myxomas usually have a stalk, are mobile, and are attached to the fossa ovalis. Intracardiac, especially atrial, thrombi are most often found in the left atrial appendage, or along the back wall of the atrium, have no stalks, and are seen with conditions such as atrial fibrillation and mitral stenosis, or with spontaneous echocardiographic contrast [13].

Differentiating myxomas from malignant tumors with echocardiography can be clinically challenging. However, atypical localization of a significantly vascularized mass during contrast echocardiography could be suggestive of a malignant cardiac tumor [14, 15, 16].

Several clinical studies have attempted to identify echocardiographic characteristics of myxomas that could predict either recurrence or a poorer prognosis. Recurrence rates are higher in younger people and when localization is atypical, but parameters related to size, shape, and site of origin have not been associated with poorer survival [17].

The limitations of echocardiography include a narrower field of view than MRI and CT, greater interobserver variation, lack of tissue characterization, restricted acoustic window in some patients (particularly those who are obese or have chronic lung disease), and ultrasound artifacts that may mimic pathology [3, 18]. Consequently, further evaluation using CT and/or MRI is a reasonable next step in the diagnostic algorithm. Multimodality imaging is particularly recommended if the interatrial septal attachment is absent, tumors are sessile, the patient has a history of cancer, there is an atypical echocardiographic presentation or a poor acoustic window, and if a thrombus is suspected [1].

## 3. Computed Tomography

Cardiac CT has become valuable for providing a more detailed analysis of cardiac masses, particularly when other imaging modalities are contraindicated or provide limited diagnostic information.

The advantages of CT include fast acquisition, high spatial and temporal resolution, multiplanar reconstructions, excellent calcification imaging, extracardiac tissue assessment, utility for preoperative planning, exclusion of obstructive coronary artery disease, and suitability for patients with contraindications for MRI [19]. The main disadvantages of CT are the use of ionizing radiation, especially if retrospective electrocardiographic (ECG) gating is applied, and the use of iodinated contrast material, which can limit CT use in patients with advanced renal failure and previous hypersensitivity reactions to these media. Compared with MRI, CT has a lower soft-tissue resolution, although this may be improved with newer spectral CT technology. CT quality may be reduced in patients with arrhythmia, those who are unable to cooperate during scanning, and those with metallic objects close to the heart (e.g., cardiac pacemakers or implantable cardioverter-defibrillators).

**Fig. 2.**
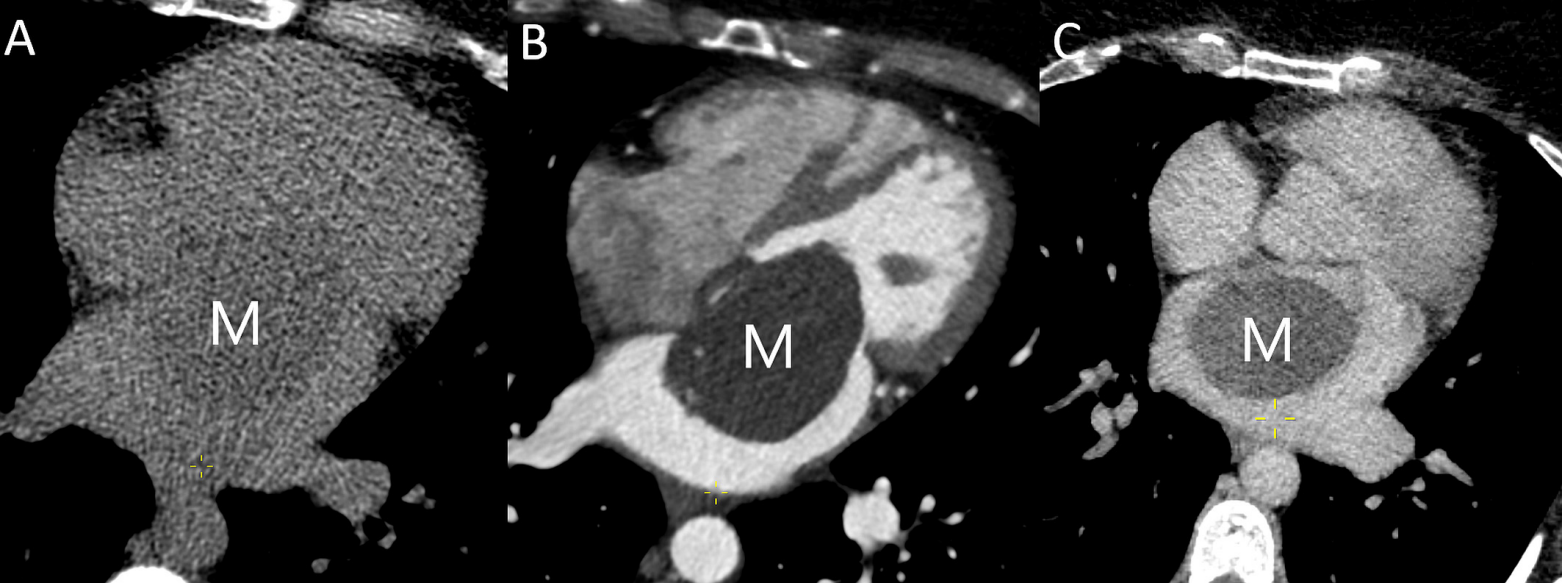
**Left atrial myxoma (M) imaged using cardiac CT.** (A) The mass is slightly hypoattenuated compared with the blood pool on the non-enhanced scan. (B) In the arterial phase, the mass appears as a large left atrial filling defect attached to the interatrial septum and protruding through the mitral valve in diastole. (C) Slight heterogeneous postcontrast enhancement in the venous phase. CT, computed tomography.

The CT protocol for assessing cardiac masses differs from that used for coronary artery imaging, although coronary arteries can be evaluated with this protocol if needed. CT images should be acquired during breath-hold with ECG gating. The usual CT protocol for evaluating a mass includes non-contrast CT to detect calcification, measurement of the precontrast attenuation values of the mass, and then postcontrast CT in the systemic arterial and venous phases to detect enhancement and detailed intra- and extracardiac anatomy (Fig. 2). This standard protocol can be modified depending on the clinical question and the location of the cardiac mass. For example, a mass in the right heart requires a shorter scanning delay when the right cardiac chambers are optimally filled with contrast. To reduce the radiation dose, prospective ECG triggering in diastole can be used in this multi-phase scanning protocol and is usually sufficient for morphological evaluation of the mass. Dynamic CT evaluation of the mass may be performed during the cardiac cycle if retrospective ECG gating is applied, for example, if there is a transmitral myxoma prolapse and consecutive valvular obstruction. However, retrospective ECG gating is rarely used because of the significantly higher radiation dose, and the possibility of evaluating mobility using other imaging techniques (echocardiography or MRI).

**Fig. 3.**
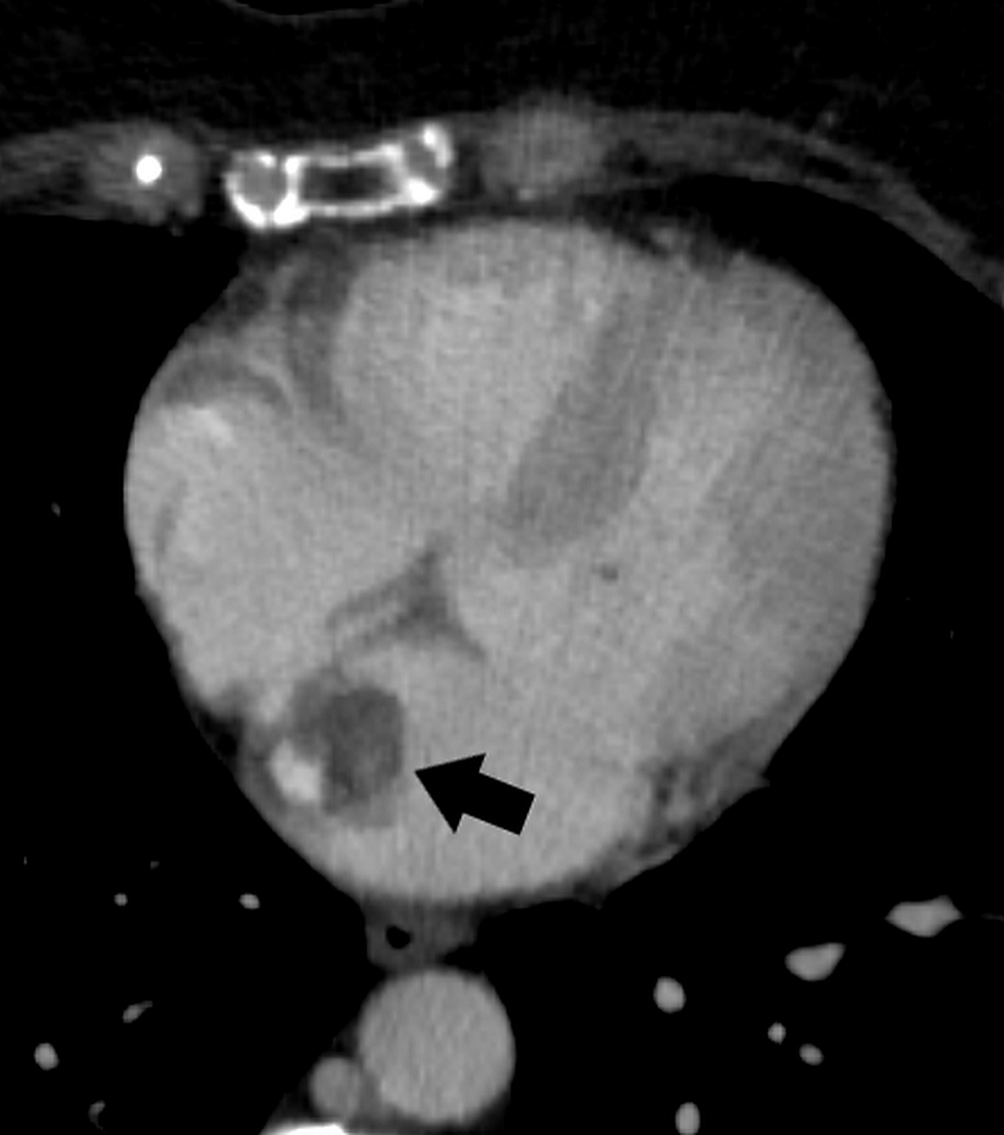
**Incidentally detected left atrial myxoma (arrow) on a chest CT of a patient with papillary thyroid cancer.** The left atrial mass was surgically removed and histologically confirmed as a myxoma. CT, computed tomography.

**Fig. 4.**
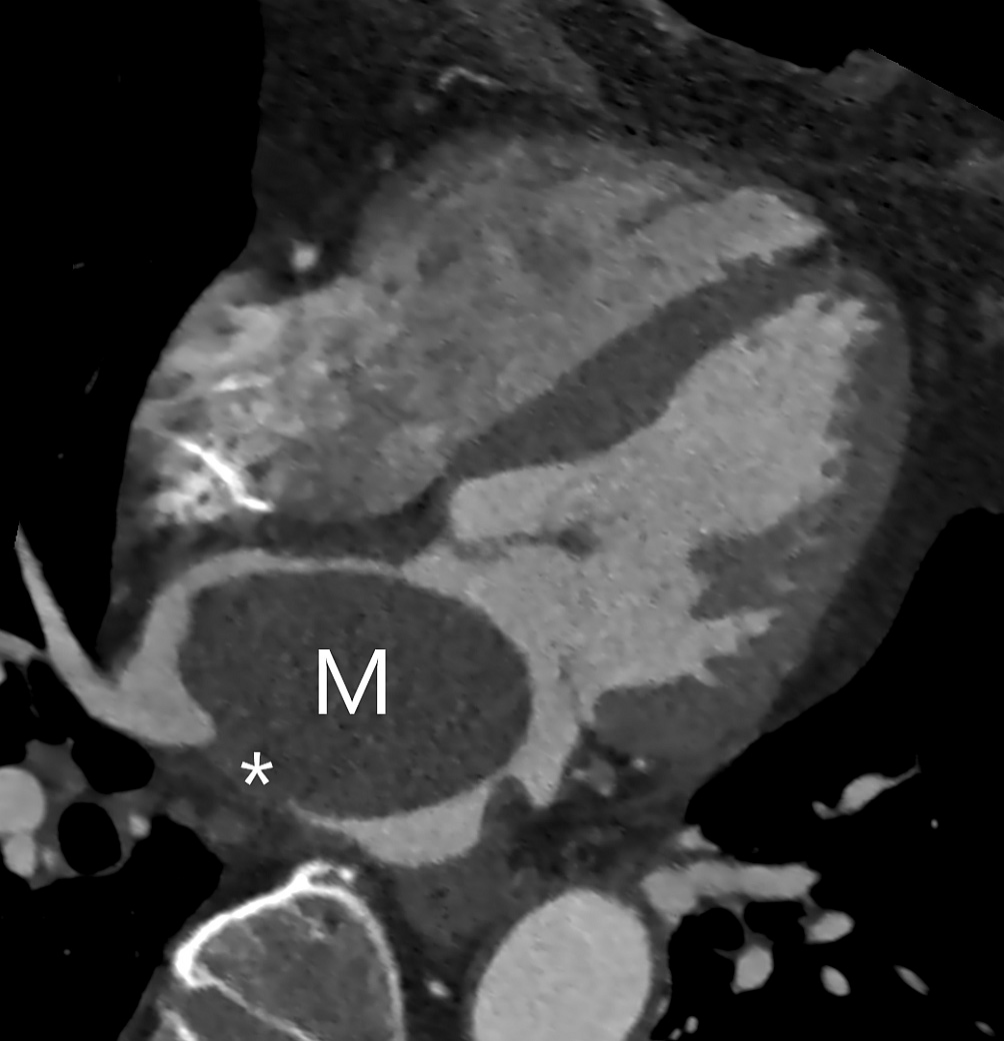
**Cardiac CT shows a left atrial myxoma (M) in a less typical location with the stalk (asterisk) attached to the left atrial posterior wall**.

**Fig. 5.**
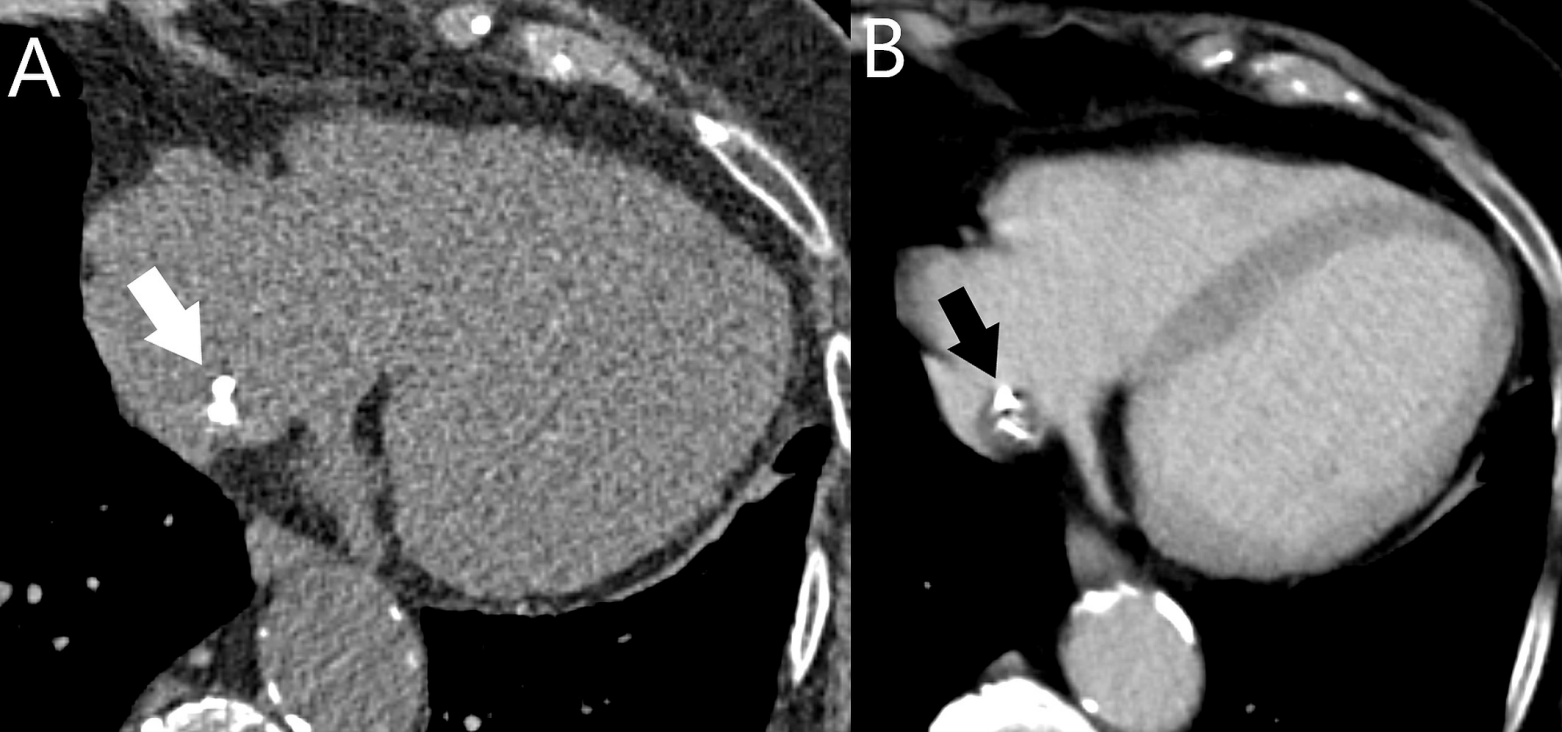
**Patient with a right atrial myxoma (arrows).** The mass is partly calcified and attached to the interatrial septum. (A) Non-enhanced cardiac CT. (B) Postcontrast cardiac CT. CT, computed tomography.

**Fig. 6.**
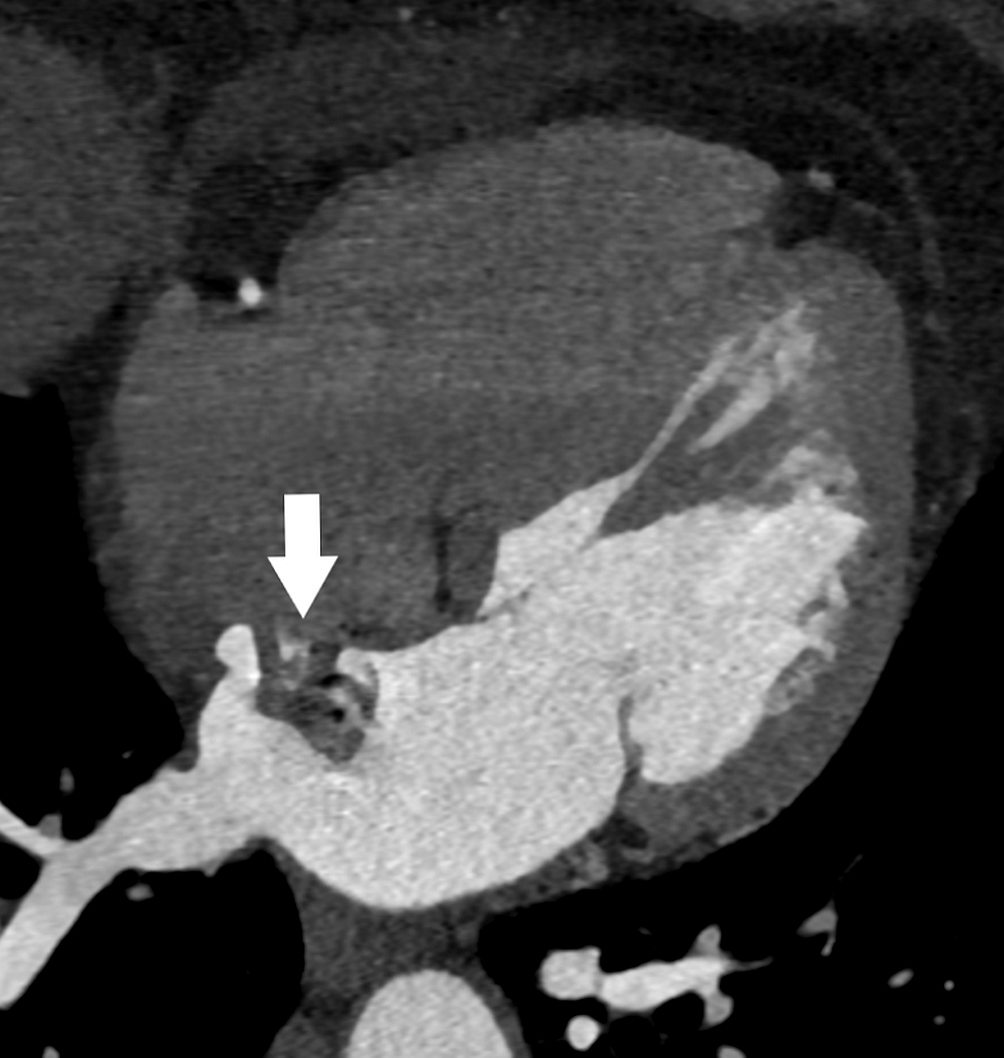
**Cardiac CT of a left atrial myxoma with a wide stalk (arrow) attached to the interatrial septum.** CT, computed tomography.

Myxomas are most frequently located in the atria, with 80% of cases found in the left atrium (Fig. 3) [1]. Left atrial myxomas are usually found next to the interatrial septum; less commonly they are attached to the left atrial roof or free wall (Fig. 4). They have mostly similar or slightly lower attenuation values than the surrounding intracardiac non-enhanced blood pool on non-contrast CT, although 10–30% contain calcifications due to previous hemorrhages (Fig. 2) [20]. Calcifications are more common in right heart myxomas (Fig. 5). Myxomas are usually ovoid with a lobulated or smooth surface; the base can be broad or have a narrow stalk, resulting in a peduncular appearance with potential mobility (Fig. 6).

On postcontrast CT, myxomas appear as intracavitary filling defects during the arterial phase with heterogeneous postcontrast enhancement on delayed phases, depending on necrosis, calcification, and thrombosis. They sometimes show only a slight increase in attenuation values after contrast injection, which may be insufficient to distinguish them from thrombi [21]. Differentiation between poorly enhanced myxomas and thrombi may be improved using dual-energy CT with iodine concentration measurement [1].

Myxomas can grow quite large and cause symptoms by moving through heart structures (often valves) and causing an obstruction (Fig. 2). In a typical left atrial myxoma case, a mobile peduncular mass may cause diastolic obstruction of the mitral valve.

## 4. Magnetic Resonance Imaging

Cardiac MRI is a noninvasive, nonionizing technique used to characterize heart anatomy, physiology, and pathology. It plays an important role in evaluating cardiovascular diseases, including ischemic heart disease, cardiomyopathy, valvular disease, congenital heart disease, pericardial disease, and cardiac masses [22]. Compared with other imaging modalities, MRI has several advantages including a uniquely high soft-tissue resolution, multiplanar acquisition capability, and the ability to visualize the heart and surrounding structures. Furthermore, protocols can be tailored to address likely differential diagnoses using a large number of available sequences, without the need for ionizing radiation or iodinated contrast media [23].

Like CT, MRI scanning should be synchronized with ECG, and respiratory gating is needed during image acquisition, usually by breath-hold techniques.

Cardiac planes are established for each patient using scout images and include long-axis images (two-chamber, four-chamber, and three-chamber views) as well as a short-axis stack of images extending from the mitral valve to the cardiac apex. Additional planes are routinely used to evaluate the heart, such as the left and right ventricular outflow views.

The MRI protocol for suspected cardiac masses typically includes T1- and T2-weighted images through the mass with and without fat suppression for tissue characterization, cine images in different cardiac planes for functional evaluation, as well as first-pass perfusion and late gadolinium enhancement (LGE) images to assess mass vascularization [24, 25, 26]. It may also be useful to acquire T1, T2, and T2* maps through the mass to improve tissue characterization. This combination of sequences allows tumor characterization and may help distinguish between malignant and benign tumors [27].

Cardiac dark-blood T1- and T2-weighted images are designed for imaging cardiovascular structures by suppressing the blood signal, thus highlighting the myocardial or vascular wall signal [28]. This involves adding radiofrequency preparation pulses to suppress the blood signal, resulting in an image with a dark-blood appearance. The fat signal can also be suppressed, resulting in an image with a dark-blood and dark-fat appearance. T1- and T2-images allow tissue characterization of the mass and the fast acquisition time minimizes respiratory and cardiac movement artifacts. It is also possible to use inversion recovery sequences like short-tau inversion recovery and turbo inversion recovery magnitude sequences, in which pulses are used to null the signal from fat and other tissues to improve contrast.

In contrast to dark-blood imaging, bright-blood imaging involves gradient echo sequences (GRE) and is more commonly used today with steady-state free precession (SSFP) sequences. The main advantage of bright-blood imaging is its fast acquisition. Cine MRI captures images of the heart in motion throughout the cardiac cycle and displays the cardiac motion in a cine loop (**Supplementary Fig. 1**). In patients with a cardiac mass, this technique allows evaluation of mass mobility and hemodynamic consequences like valvular obstruction. However, cine images are prone to artifacts in patients with cardiac arrhythmias, and it may be difficult to follow the tumor motion during the cardiac cycle, particularly if the mass is small.

First-pass perfusion imaging allows dynamic evaluation of contrast enhancement of the mass using fast, fat-saturated GRE, SSFP, or echo-planar imaging with images acquired either every heartbeat or every second beat [29]. It requires fast intravenous injection of gadolinium-based contrast agents, which are followed through the right cardiac chambers and left cardiac chambers, finally reaching the myocardium and other tissues including the cardiac mass. Myxomas typically show poor early contrast enhancement with heterogeneous enhancement later after contrast injection (**Supplementary Fig. 2**).

**Fig. 7.**
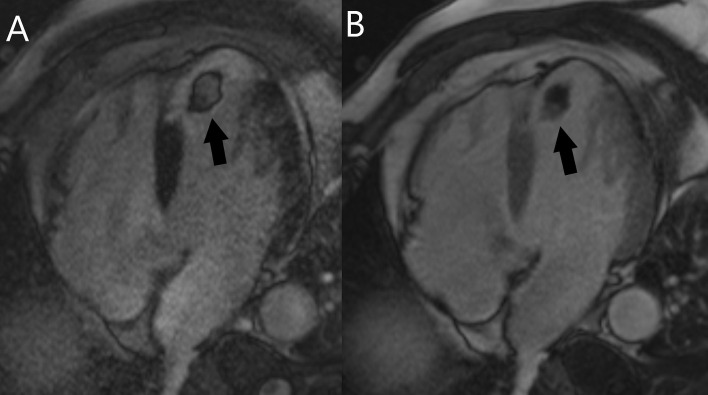
**MRI late-gadolinium enhancement in a patient with myocardial infarction and a thrombus (arrows) in the left ventricular apex.** (A) Standard inversion time (280 msec at 1.5 Tesla) nulls the signal of viable myocardium. (B) The avascular cardiac thrombus appears black if a long inversion time is applied (600 msec at 1.5 Tesla) enabling its differentiation from vascularized cardiac masses. MRI, magnetic resonance imaging.

Postcontrast LGE images are obtained approximately 10 minutes after contrast application using a T1-weighted rapid GRE sequence combined with an inversion-recovery pre-pulse to null the signal from the normal myocardium. Focal myocardial fibrosis exhibits a delayed gadolinium contrast washout, so hyperenhancement indicates a myocardial scar. It is used to distinguish ischemic from non-ischemic cardiomyopathy based on different enhancement patterns. During cardiac mass evaluation, it is used to assess the presence and pattern of postcontrast enhancement and tumor vascularity and to differentiate cardiac masses from avascular cardiac thrombi. Intracavitary masses should be assessed with long inversion time LGE imaging (approximately 600 msec at 1.5 Tesla and 875 msec at 3 Tesla) to distinguish between tumor and thrombus (Fig. 7) [30]. The signal from the thrombus is maximally nulled much later than signals from other tissues and is readily distinguished using this technique.

**Fig. 8.**
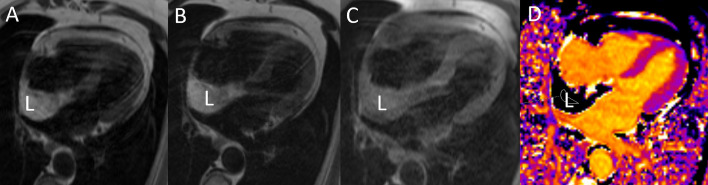
**MRI tissue characterization in a patient with lipomatous hypertrophy of the interatrial septum (L).** (A) The interatrial septum is thickened and has a bright signal on the T1-weighted image. (B) A bright signal is also seen on the T2-weighted image. (C) There is signal suppression on the T1-weighted fat-suppressed image. (D) T1 mapping shows a very low T1 relaxation time (187 msec at 1.5 Tesla). MRI, magnetic resonance imaging.

Multiparametric cardiac MRI using T1, T2, and T2* mapping allows direct measurement of relaxation times in tissues allowing advanced tissue characterization. Low T1 relaxation times are found in fat tissue (Fig. 8) and if iron has been deposited, for example following intratumoral or intramyocardial hemorrhage. There is a long T1 relaxation time in tissues with enlarged extracellular spaces, such as fibrotic tissue. Extracellular volume fraction can be estimated by measuring myocardial and blood T1 relaxation time before and after contrast administration if the patient’s hematocrit value is available [31]. T2 mapping helps detect edema in cases of acute myocardial infarction, myocarditis, or graft rejection, and when cardiac masses have a high water content. T2* mapping can be useful for cardiac thrombi and intratumoral hemorrhage, which are represented by low T1 and T2* relaxation times [32].

**Fig. 9.**
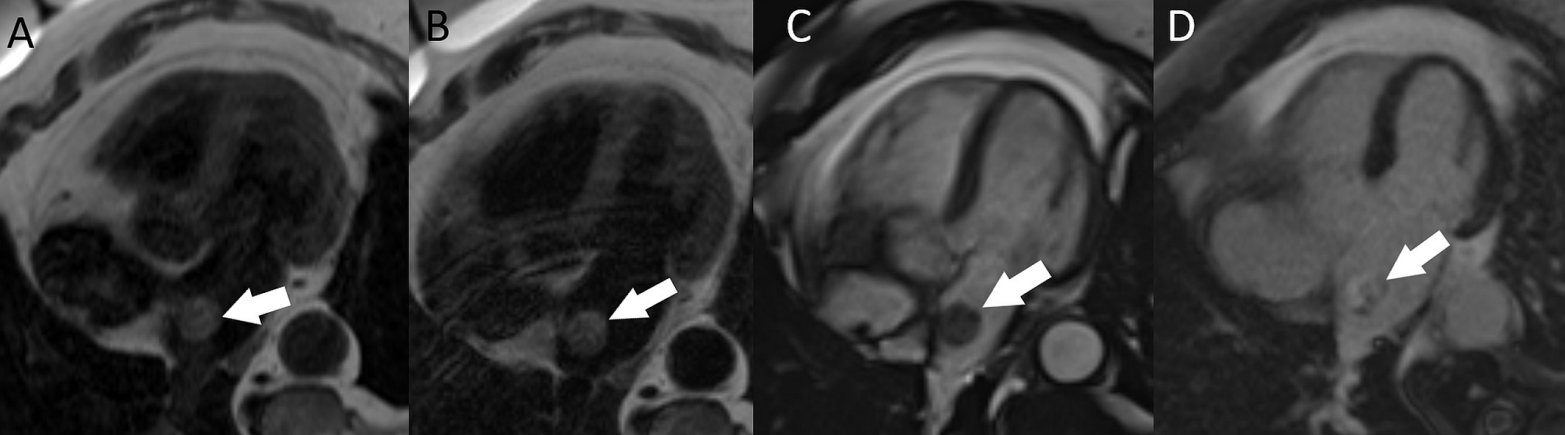
**Cardiac MRI in a patient with left atrial myxoma (arrow).** (A) The mass is isointense to the myocardium on T1-weighted images. (B) The mass is slightly hyperintense on T2-weighted images. (C) On SSFP images it has a higher signal than the myocardium and a lower signal than the blood pool. (D) Heterogeneous late gadolinium enhancement of the mass. MRI, magnetic resonance imaging; SSFP, steady-state free precession.

**Fig. 10.**
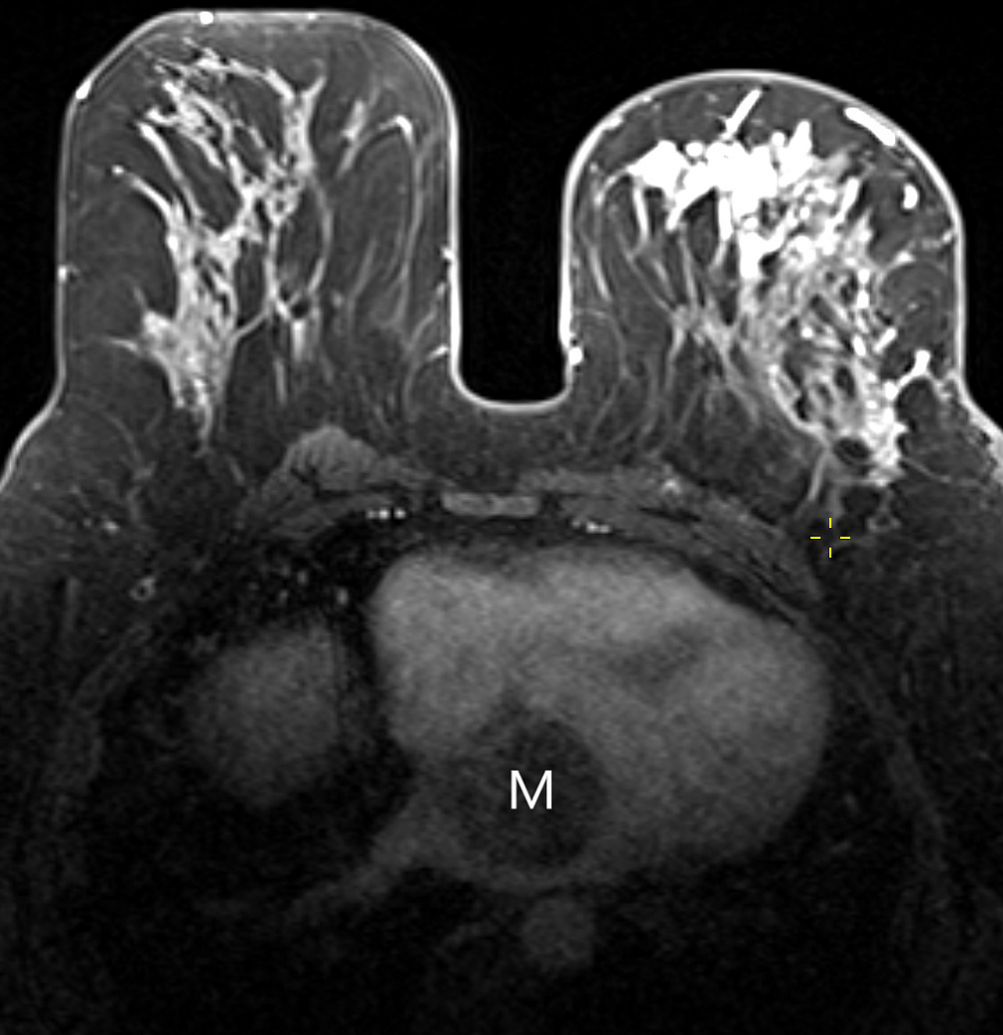
**Incidentally detected left atrial myxoma (M) on breast MRI in a patient with a newly diagnosed invasive breast cancer.** The left atrial mass was surgically removed and histologically confirmed as a myxoma. MRI, magnetic resonance imaging.

Cardiac MRI provides detailed views of cardiac morphology, aiding the visualization of intracardiac masses like myxomas and allowing comprehensive assessment of tumor location, size, attachment points, and potential invasion of adjacent cardiac structures. The myxoma is most frequently visualized as a mass isointense to the myocardium on T1-weighted imaging, and hyperintense on T2-weighted imaging with foci of hypointensity in one or two of these sequences (Fig. 9) [33]. On SSFP sequences, myxomas have a higher signal than the myocardium and a lower signal than the bright-blood pool [26]. They are typically located in the atria, particularly the left. Cardiac myxomas often exhibit heterogeneous signal intensity on different sequences, typically appearing as a mass with variable signal intensity due to their gelatinous composition and the mixture of cells, collagen, and mucopolysaccharides. Contrast-enhanced MRI allows excellent definition of the tumor’s vascularity, helping to differentiate it from other cardiac masses or thrombi. Additionally, functional assessment can help assess the tumor’s potential impact on cardiac function and blood flow dynamics, such as diastolic mitral valve obstruction by a left atrial myxoma. A cardiac myxoma may also be detected incidentally during other MRI examinations, such as a chest or breast MRI (Fig. 10).

The main disadvantages of MRI include the longer acquisition time compared with CT, lower availability compared with other imaging techniques, and contraindications in patients with claustrophobia and those with ferromagnetic foreign bodies and older cardiac devices [18].

## 5. Differential Diagnosis

Myxomas are the most common benign cardiac masses. They are easily identified when located in the atria, particularly the left atrium, when attached to the interatrial septum. Less common locations include the posterior or lateral left atrial free wall (Fig. 4), mitral or tricuspid valve, left atrial appendage, posterior right atrial wall, and, rarely, the right or left ventricle or pulmonary artery [1, 34]. Right atrial myxomas are more common in children than in adults [18]. Biatrial involvement may occur if the myxoma grows across the patent foramen ovale. Multifocal myxomas in atypical sites have been reported in patients with Carney’s complex [26, 35].

**Fig. 11.**
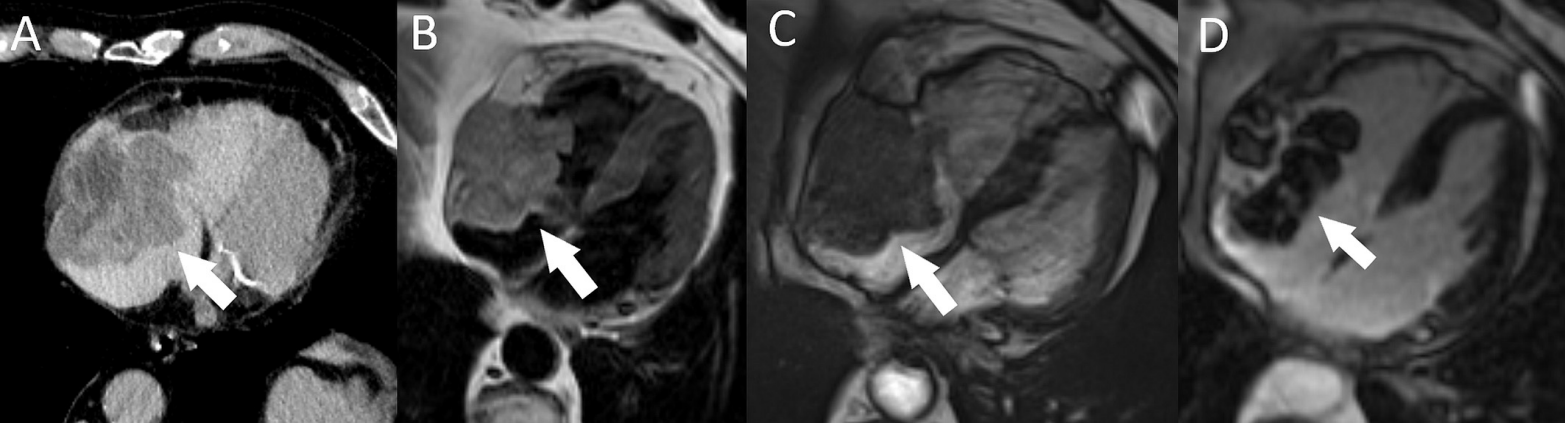
**Right atrial metastasis (arrow) in a patient with metastatic melanoma.** (A) A heterogeneous, partially necrotic mass with invasion of the right atrial free wall and epicardial fat tissue seen on postcontrast chest CT. (B) T2-weighted cardiac MRI. (C) SSFP MRI. (D) LGE MRI. CT, computed tomography; MRI, magnetic resonance imaging; LGE, late gadolinium enhancement; SSFP, steady-state free precession.

Metastatic malignant tumors are 22 to 132 times more common than primary cardiac masses and should be considered in every patient with disseminated malignancy [18]. The most common malignancies with cardiac metastases are melanoma and breast and lung cancers (Fig. 11). Metastases may be multiple and located anywhere in the heart. Hemorrhagic pericardial effusion is commonly seen in patients with pericardial metastases.

**Fig. 12.**
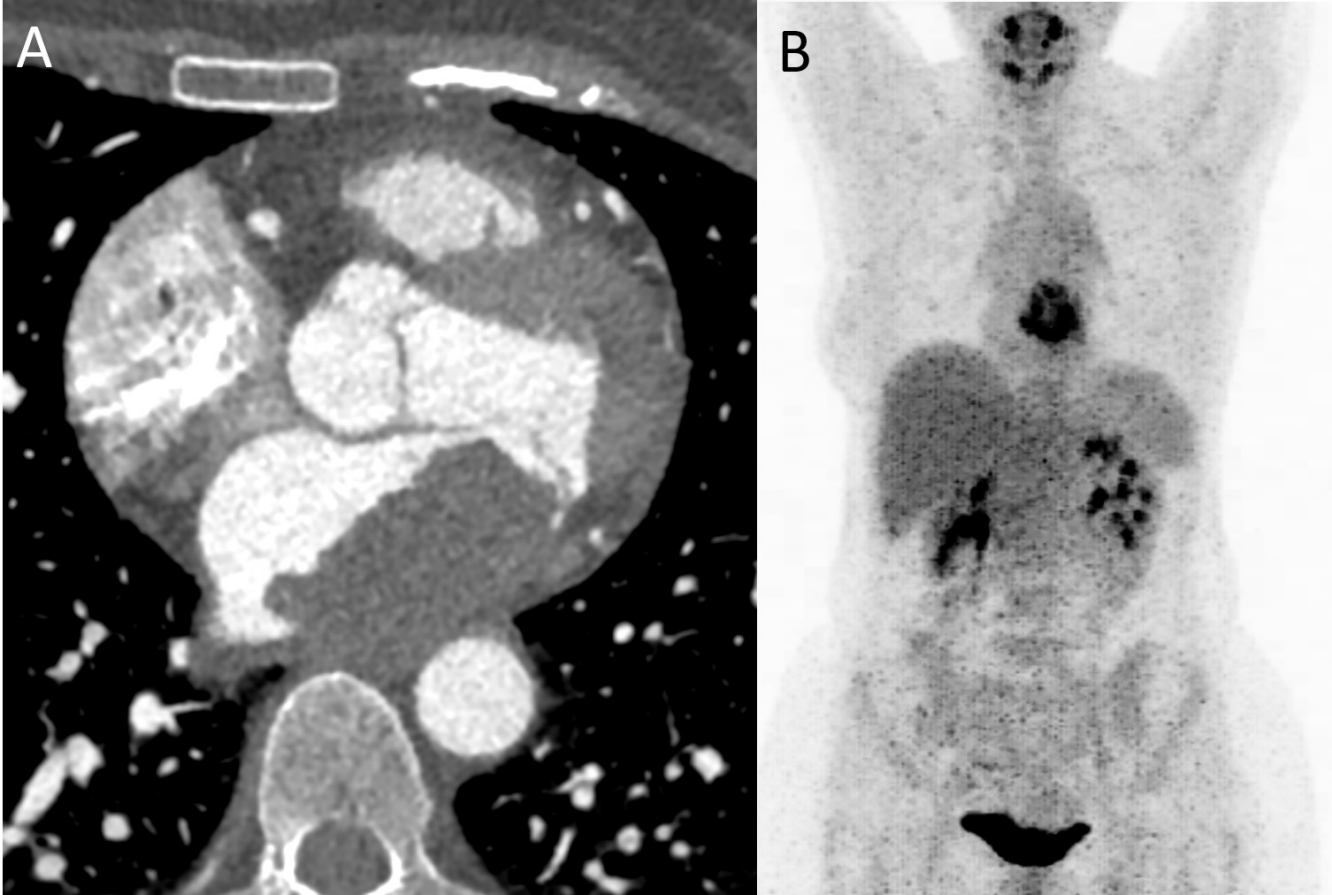
**Imaging of a patient with angiosarcoma.** (A) Cardiac CT shows a histologically proven left atrial angiosarcoma (arrow) with tumor invasion of mediastinal fat. (B) PET-CT shows high 18F-FDG uptake into the mass. CT, computed tomography; 18F-FDG, 18F-fluorodeoxyglucose; PET-CT, positron-emission tomography with computed tomography.

Angiosarcoma is the most common primary heart malignancy. It is more common in the right cardiac chambers and has malignant morphological characteristics, including intratumor heterogeneity and invasion of adjacent structures with or without pericardial effusion (Fig. 12). Hemorrhagic pericardial effusion in a patient with a cardiac mass is highly suspicious of malignancy. Positron emission tomography with CT (PET-CT) can be used to assess both primary and metastatic cardiac malignancies. PET-CT shows the metabolic activity of cardiac tumors with 18F-fluorodeoxyglucose (18F-FDG), along with inflammatory conditions like sarcoidosis, infection, and postsurgical or postradiation changes, as well as brown fat [36]. However, optimal assessment of 18F-FDG uptake into the heart requires suppression of normal myocardial glucose utilization with a high-fat, low-carbohydrate diet for at least two meals and a ≥4-hour fast before scanning [37].

**Fig. 13.**
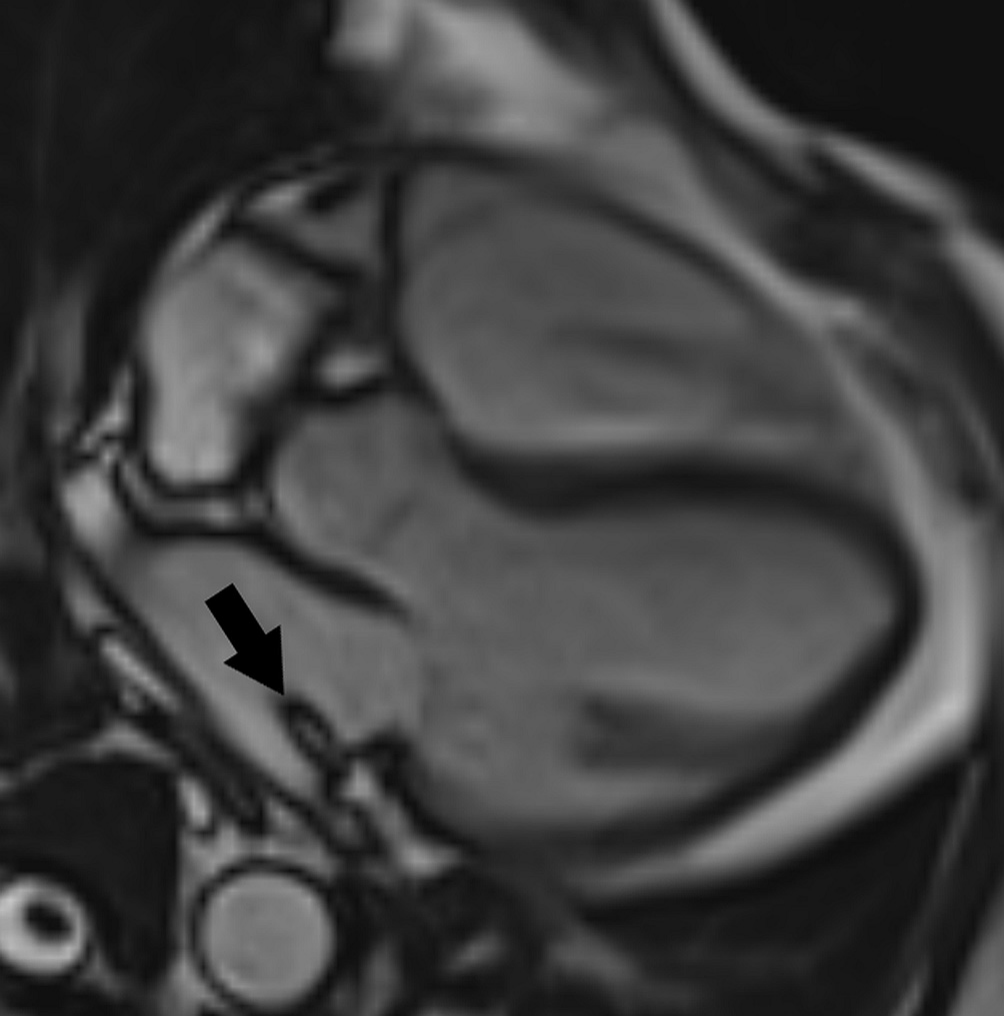
**The coumadin ridge (arrow) is a lateral fold of left atrial wall tissue between the left atrial appendage and the left superior pulmonary vein (SSFP image).** SSFP, steady-state free precession.

**Fig. 14.**
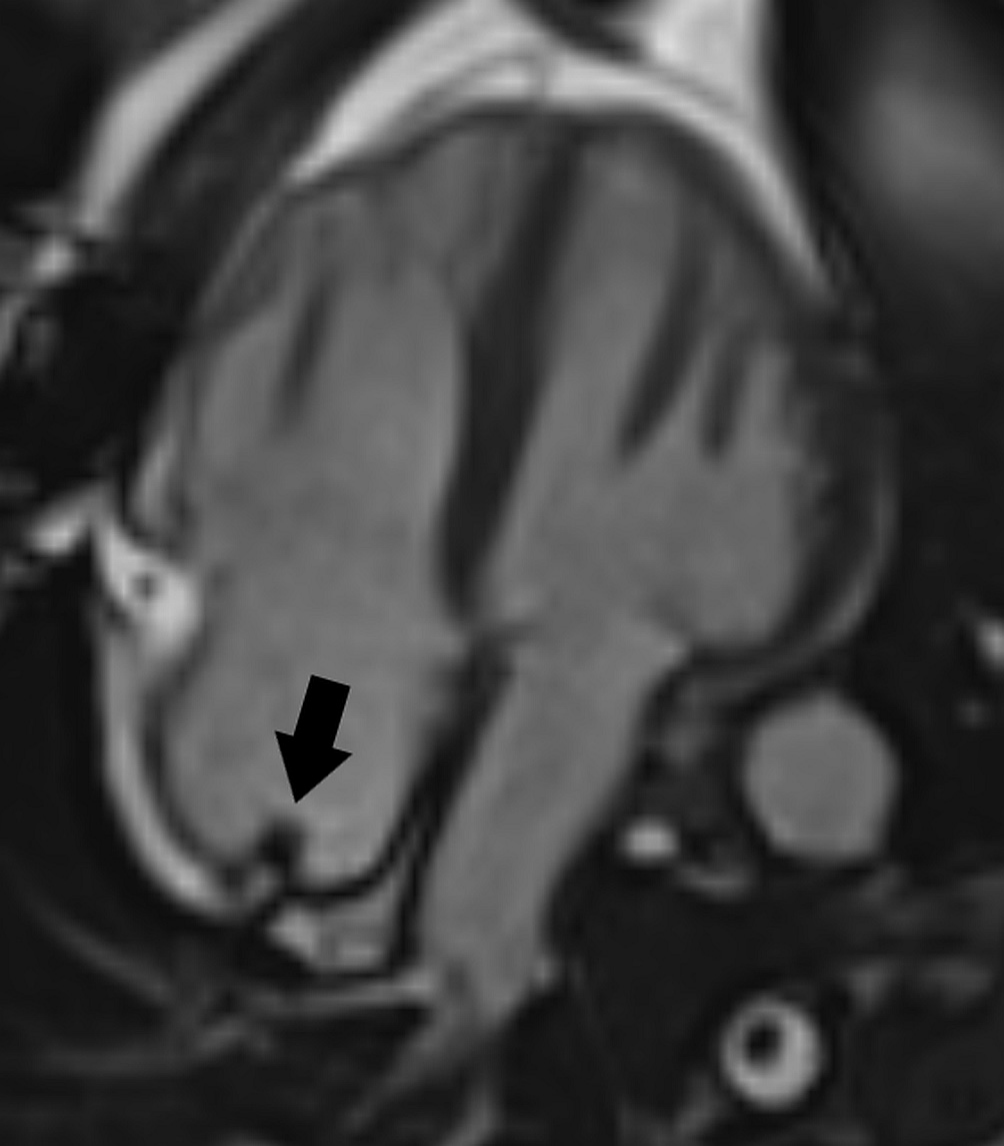
**The crista terminalis (arrow) is a muscular ridge in the right atrium extending between the orifices of the superior and inferior vena cavae that separate the trabeculated and non-trabeculated regions of the right atrium**.

Several normal anatomical structures mimic cardiac masses, including the coumadin ridge (Fig. 13) in the left atrium separating the left superior pulmonary vein and left atrial appendage, as well as the crista terminalis (Fig. 14), which separates the trabeculated and non-trabeculated regions of the right atrium and extends from the orifice of the superior vena cava to that of the inferior vena cava [38, 39].

**Fig. 15.**
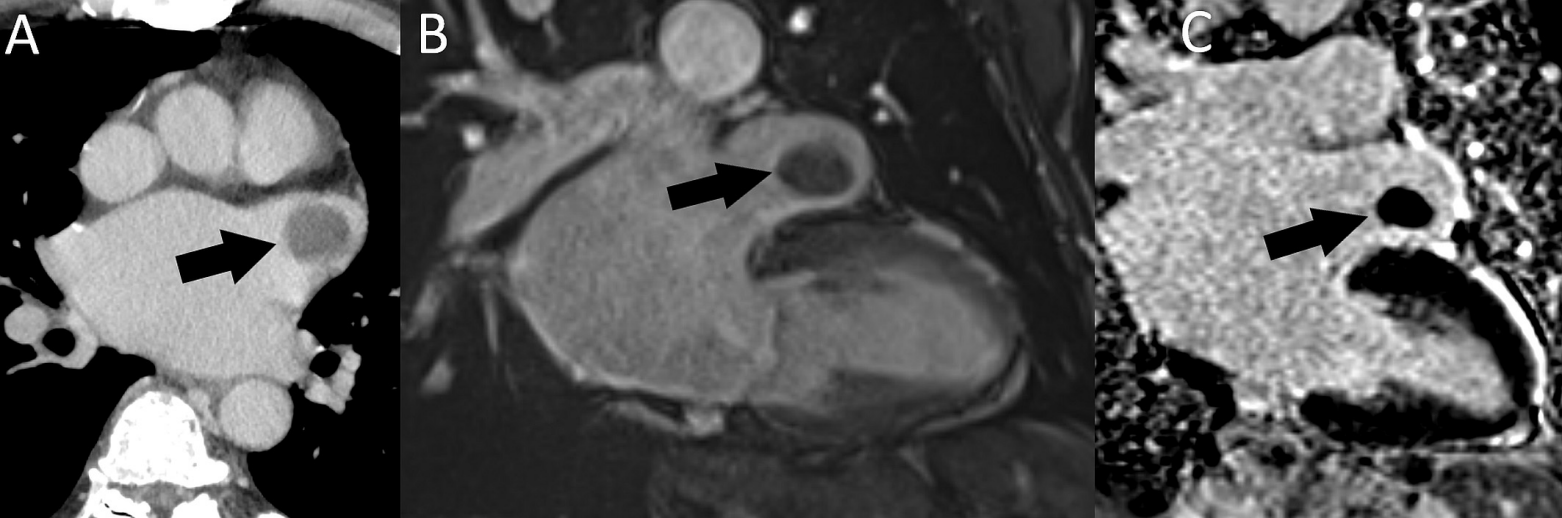
**Thrombus (arrow) in a dilated left atrial appendage.** (A) Typical location on contrast-enhanced CT. (B) Two-chamber view SSFP sequence. (C) The thrombus is avascular and black on a phase-sensitive inversion recovery LGE image. CT, computed tomography; LGE, late gadolinium enhancement; SSFP, steady-state free precession.

Cardiac thrombi are the most common non-neoplastic cardiac masses that may be mistaken for tumors. Thrombi are typically sessile and are found in the left atrial appendage (Fig. 15), particularly in patients with atrial fibrillation or mitral valve disease. They are also found in the hypokinetic left ventricular apex after myocardial infarction or apical aneurysm, in cardiac aneurysms and pseudoaneurysms at other sites, and in sites with slow blood flow. Right atrial thrombi most commonly occur in patients with central venous catheters or cardiac pacemakers. In addition to their location, thrombi can be differentiated from cardiac neoplasms by their avascularity and lack of enhancement on delayed postcontrast CT images or on early and late gadolinium enhancement images on MRI. Therefore, there is no change in T1 relaxation time in thrombi after contrast administration, and they appear black if a long inversion time is used (600 msec at 1.5 Tesla and 875 msec at 3 Tesla, Fig. 7), while vascularized tissues will appear grey or white [25, 30]. Rarely, slight peripheral enhancement may be observed in highly organized and very chronic thrombi [26]. Another important imaging feature is that a thrombus is less mobile than a myxoma and rarely causes dynamic atrioventricular valvule obstruction [21]. In addition, cardiac thrombi have short T1 and T2* relaxation times on multiparametric cardiac MRI [32]. Confident diagnosis of cardiac thrombi is important because affected patients require anticoagulant therapy. Thrombi may also form on the surface of polypoid and extensively myxoid myxomas, or those with an irregular papillary surface with subsequent risk of embolization [3].

**Fig. 16.**
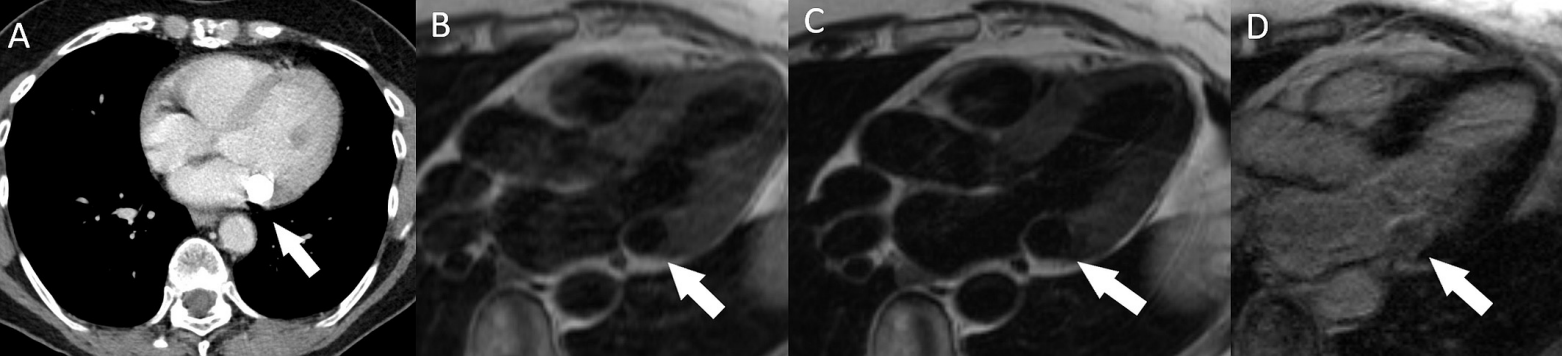
**Caseous calcification of the mitral valve (arrow).** (A) On CT there is a calcified mass in the posterior part of the mitral annulus. (B) On cardiac MRI the mass has a low signal on a T1-weighted image. (C) A low signal is also seen on a T2-weighted image. (D) Peripheral LGE. CT, computed tomography; MRI, magnetic resonance imaging; LGE, late gadolinium enhancement.

Caseous calcification of the mitral annulus is another non-tumorous mass found in the left atrium. It is oval, round, or semicircular, and is typically located in the posterior part of the mitral annulus (Fig. 16). On non-contrast CT it appears peripherally calcified with a hyperdense material in the center and no contrast enhancement [40]. On MRI, it has a low signal on T1- and T2-weighted images and cine SSFP imaging, without enhancement during first-pass perfusion imaging. Peripheral LGE of the mass can be observed [41].

**Fig. 17.**
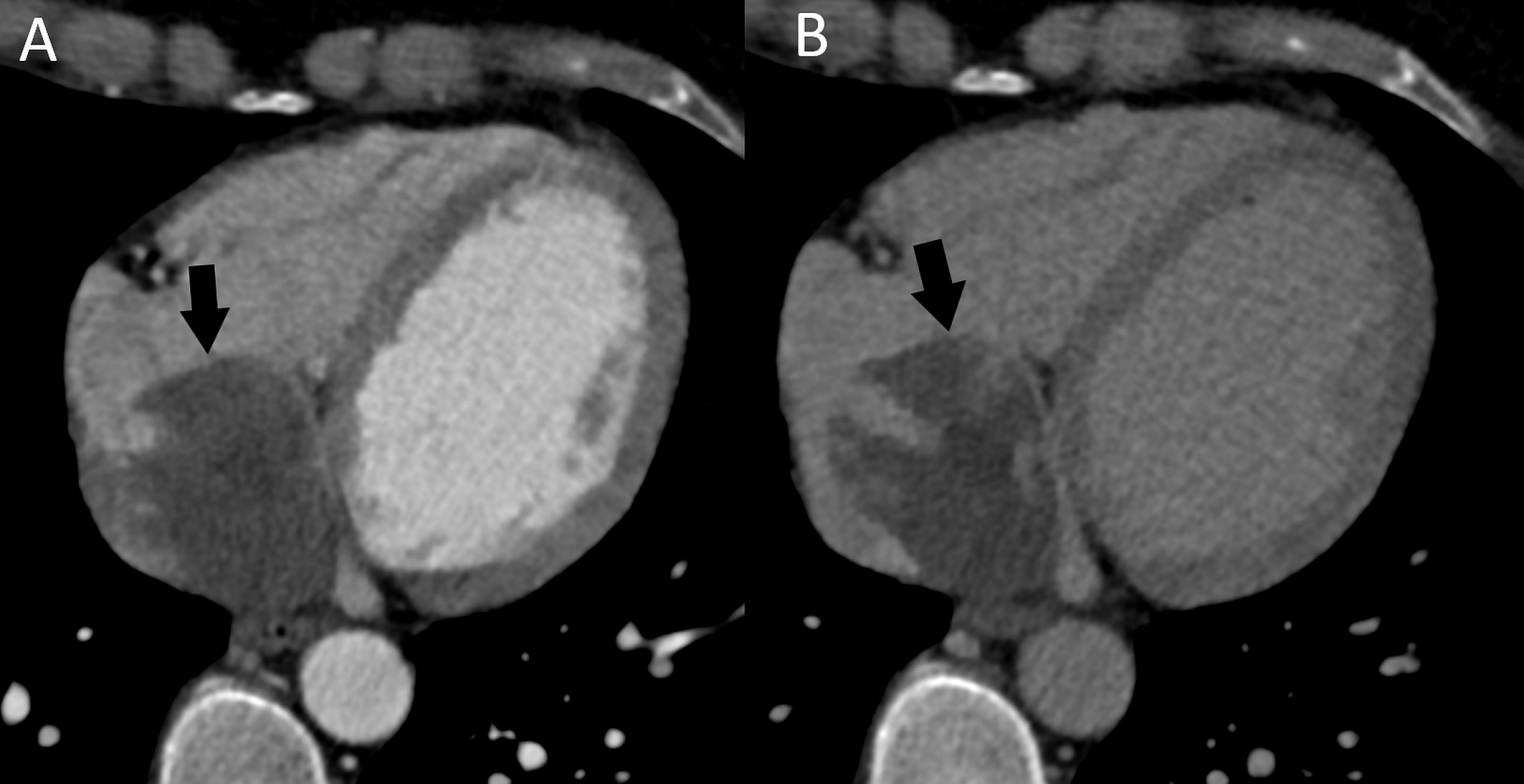
**Right atrial hemangioma (arrow).** (A) A peripheral nodular enhancement is seen on the arterial phase CT. (B) Slow filling in the venous and delayed images. CT, computed tomography.

**Fig. 18.**
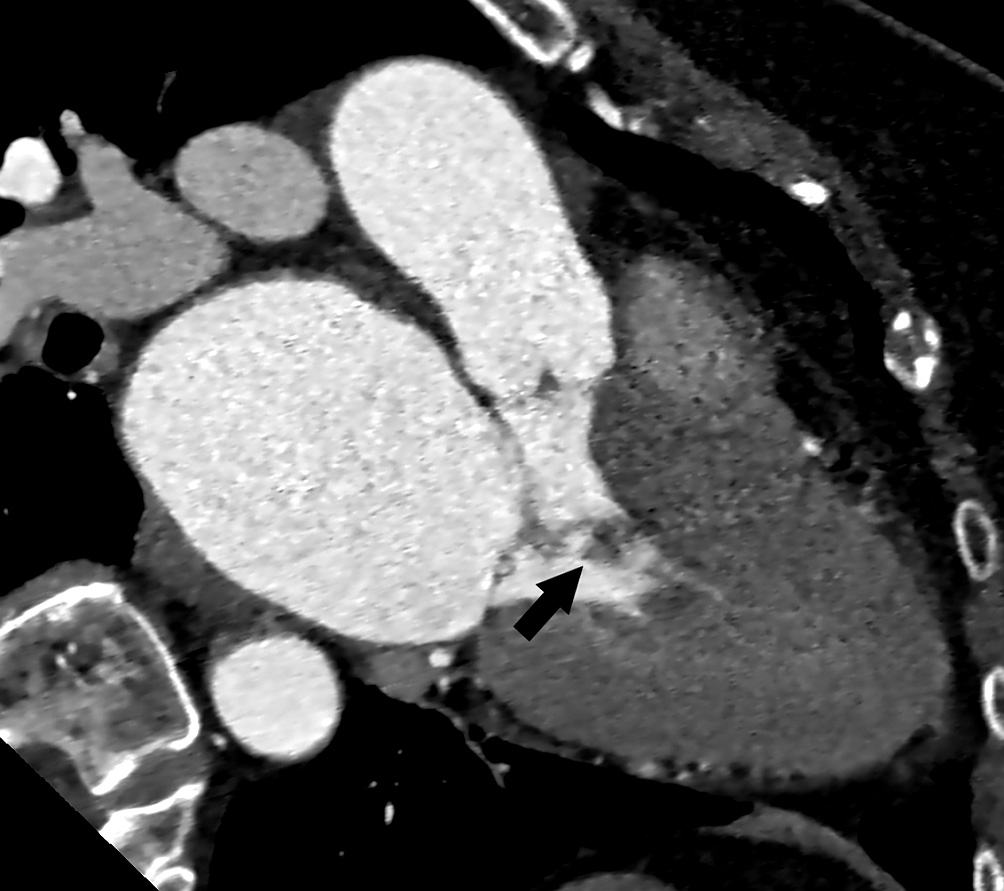
**Papillary fibroelastoma (arrow) of the mitral subvalvular apparatus in a patient with hypertrophic cardiomyopathy on a three-chamber CT reformatted image.** CT, computed tomography.

Other benign neoplastic cardiac masses that can mimic a myxoma include cardiac lipoma and hemangioma. Cardiac lipomas can easily be identified by fat-tissue attenuation on CT and a high signal on T1- and T2-weighted MRI, with a loss of signal if a fat-suppression sequence is applied. Lipomatous hypertrophy of the interatrial septum has identical tissue characteristics but is typically located in the interatrial septum, and has a dumb-bell shape whilst sparing of the fossa ovalis (Fig. 8). Cardiac hemangiomas have a high signal intensity on T2-weighted images like myxomas, but have an early peripheral nodular enhancement after contrast administration with filling in on delayed images (Fig. 17) [42]. Like myxomas, papillary fibroelastomas arise from the endocardium but are usually small and attached to cardiac valves and enhance more homogeneously than myxomas (Fig. 18) [26].

## 6. Conclusions

Multimodality cardiac imaging provides reliable information on the location, size, and tissue characteristics of cardiac myxomas and other cardiac masses, and is a key source of data for treatment planning. To optimize their utilization, it is important to be aware of the advantages and limitations associated with all imaging techniques, including echocardiography, cardiac CT, and MRI. Correct diagnosis of cardiac myxomas allows timely surgical treatment and can prevent embolic events, heart failure, and sudden cardiac death.

## Abbreviations

CT, computed tomography; 18F-FDG, 18F-fluorodeoxyglucose; ECG, electrocardiography; GRE, gradient echo sequences; LGE, late gadolinium enhancement; MRI, magnetic resonance imaging; PET-CT, positron emission tomography with computed tomography; SSFP, steady-state free precession.

## Supplementary Material

Supplementary material associated with this article can be found, in the online version, at https://doi.org/10.31083/j.rcm2506204.

## References

[1] Colin GC, Gerber BL, Amzulescu M, Bogaert J (2018). Cardiac myxoma: a contemporary multimodality imaging review. *The International Journal of Cardiovascular Imaging*.

[2] Mügge A, Daniel WG, Haverich A, Lichtlen PR (1991). Diagnosis of noninfective cardiac mass lesions by two-dimensional echocardiography. Comparison of the transthoracic and transesophageal approaches. *Circulation*.

[3] McAllister BJ (2020). Multi Modality Imaging Features of Cardiac Myxoma. *Journal of Cardiovascular Imaging*.

[4] Reynen K (1995). Cardiac myxomas. *The New England Journal of Medicine*.

[5] Javed A, Zalawadiya S, Kovach J, Afonso L (2014). Aortic valve myxoma at the extreme age: a review of literature. *BMJ Case Reports*.

[6] Obeid AI, Marvasti M, Parker F, Rosenberg J (1989). Comparison of transthoracic and transesophageal echocardiography in diagnosis of left atrial myxoma. *The American Journal of Cardiology*.

[7] Gadhinglajkar S, Sreedhar R (2008). Utility of transoesophageal echocardiography during surgery on left atrial myxoma. *Annals of Cardiac Anaesthesia*.

[8] Srivastava R, Hsiung MC, Fadel A, Nanda NC (2004). Transesophageal echocardiographic demonstration of biatrial myxoma. *Echocardiography (Mount Kisco, N.Y.)*.

[9] Pérez de Isla L, de Castro R, Zamorano JL, Almería C, Moreno R, Moreno M (2002). Diagnosis and treatment of cardiac myxomas by transesophageal echocardiography. *The American Journal of Cardiology*.

[10] Tolstrup K, Shiota T, Gurudevan S, Luthringer D, Luo H, Siegel RJ (2011). Left atrial myxomas: correlation of two-dimensional and live three-dimensional transesophageal echocardiography with the clinical and pathologic findings. *Journal of the American Society of Echocardiography: Official Publication of the American Society of Echocardiography*.

[11] L’Angiocola PD, Donati R (2020). Cardiac Masses in Echocardiography: A Pragmatic Review. *Journal of Cardiovascular Echography*.

[12] Engberding R, Daniel WG, Erbel R, Kasper W, Lestuzzi C, Curtius JM (1993). Diagnosis of heart tumours by transoesophageal echocardiography: a multicentre study in 154 patients. European Cooperative Study Group. *European Heart Journal*.

[13] Uenishi EK, Caldas MA, Saroute ANR, Tsutsui JM, Piotto GHM, Falcão SNRS (2008). Contrast echocardiography for the evaluation of tumors and thrombi. *Arquivos Brasileiros De Cardiologia*.

[14] Bhattacharyya S, Khattar R, Senior R (2013). Characterisation of intra-cardiac masses by myocardial contrast echocardiography. *International Journal of Cardiology*.

[15] Kirkpatrick JN, Wong T, Bednarz JE, Spencer KT, Sugeng L, Ward RP (2004). Differential diagnosis of cardiac masses using contrast echocardiographic perfusion imaging. *Journal of the American College of Cardiology*.

[16] Lee SH, Park JS, Park JH, Chin JY, Yoon WS, Kim HY (2020). Comparison of Clinical and Echocardiographic Characteristics between Cardiac Myxomas and Masses Mimicking Myxoma. *Korean Circulation Journal*.

[17] Yu L, Gu T, Shi E (2016). Echocardiographic Findings and Clinical Correlation With Cardiac Myxoma. *JACC. Cardiovascular Imaging*.

[18] Tyebally S, Chen D, Bhattacharyya S, Mughrabi A, Hussain Z, Manisty C (2020). Cardiac Tumors: *JACC CardioOncology* State-of-the-Art Review. *JACC. CardioOncology*.

[19] Kassop D, Donovan MS, Cheezum MK, Nguyen BT, Gambill NB, Blankstein R (2014). Cardiac Masses on Cardiac CT: A Review. *Current Cardiovascular Imaging Reports*.

[20] Grebenc ML, Rosado de Christenson ML, Burke AP, Green CE, Galvin JR (2000). Primary cardiac and pericardial neoplasms: radiologic-pathologic correlation. *Radiographics: a Review Publication of the Radiological Society of North America, Inc*.

[21] Scheffel H, Baumueller S, Stolzmann P, Leschka S, Plass A, Alkadhi H (2009). Atrial myxomas and thrombi: comparison of imaging features on CT. *AJR. American Journal of Roentgenology*.

[22] Rajiah PS, François CJ, Leiner T (2023). Cardiac MRI: State of the Art. *Radiology*.

[23] Ginat DT, Fong MW, Tuttle DJ, Hobbs SK, Vyas RC (2011). Cardiac imaging: Part 1, MR pulse sequences, imaging planes, and basic anatomy. *AJR. American Journal of Roentgenology*.

[24] Sparrow PJ, Kurian JB, Jones TR, Sivananthan MU (2005). MR imaging of cardiac tumors. *Radiographics: a Review Publication of the Radiological Society of North America, Inc*.

[25] Hoey ETD, Shahid M, Ganeshan A, Baijal S, Simpson H, Watkin RW (2014). MRI assessment of cardiac tumours: part 1, multiparametric imaging protocols and spectrum of appearances of histologically benign lesions. *Quantitative Imaging in Medicine and Surgery*.

[26] Abbas A, Garfath-Cox KAG, Brown IW, Shambrook JS, Peebles CR, Harden SP (2015). Cardiac MR assessment of cardiac myxomas. *The British Journal of Radiology*.

[27] Syed IS, Feng D, Harris SR, Martinez MW, Misselt AJ, Breen JF (2008). MR imaging of cardiac masses. *Magnetic Resonance Imaging Clinics of North America*.

[28] Tseng WYI, Su MYM, Tseng YHE (2016). Introduction to Cardiovascular Magnetic Resonance: Technical Principles and Clinical Applications. *Acta Cardiologica Sinica*.

[29] Saeed M, Van TA, Krug R, Hetts SW, Wilson MW (2015). Cardiac MR imaging: current status and future direction. *Cardiovascular Diagnosis and Therapy*.

[30] Jenista ER, Wendell DC, Azevedo CF, Klem I, Judd RM, Kim RJ (2023). Revisiting how we perform late gadolinium enhancement CMR: insights gleaned over 25 years of clinical practice. *Journal of Cardiovascular Magnetic Resonance: Official Journal of the Society for Cardiovascular Magnetic Resonance*.

[31] Haaf P, Garg P, Messroghli DR, Broadbent DA, Greenwood JP, Plein S (2016). Cardiac T1 Mapping and Extracellular Volume (ECV) in clinical practice: a comprehensive review. *Journal of Cardiovascular Magnetic Resonance: Official Journal of the Society for Cardiovascular Magnetic Resonance*.

[32] Sakatani Y, Kanzaki Y, Hoshiga M (2022). Left ventricular thrombus: insights into performance characteristics using T1 and T2 star cardiovascular magnetic resonance. *European Heart Journal. Case Reports*.

[33] Colin GC, Dymarkowski S, Gerber B, Michoux N, Bogaert J (2016). Cardiac myxoma imaging features and tissue characteristics at cardiovascular magnetic resonance. *International Journal of Cardiology*.

[34] Buckley O, Madan R, Kwong R, Rybicki FJ, Hunsaker A (2011). Cardiac masses, part 2: key imaging features for diagnosis and surgical planning. *AJR. American Journal of Roentgenology*.

[35] Patibandla S, Brito D, Sloyer D, Cook C, Badhwar V, Mills JD (2021). Multimodality Cardiac Imaging Enhances Diagnosis and Management of Recurrent Atrial Myxomas in Carney Complex. *CASE (Philadelphia, Pa.)*.

[36] Aggeli C, Dimitroglou Y, Raftopoulos L, Sarri G, Mavrogeni S, Wong J (2020). Cardiac Masses: The Role of Cardiovascular Imaging in the Differential Diagnosis. *Diagnostics (Basel, Switzerland)*.

[37] Osborne MT, Hulten EA, Murthy VL, Skali H, Taqueti VR, Dorbala S (2017). Patient preparation for cardiac fluorine-18 fluorodeoxyglucose positron emission tomography imaging of inflammation. *Journal of Nuclear Cardiology: Official Publication of the American Society of Nuclear Cardiology*.

[38] Lakhani DA, Balar AB, Kim C (2022). Prominent crista terminalis mimicking a right atrial mass: A case report and brief review of the literature. *Radiology Case Reports*.

[39] Silbiger JJ (2019). The Anatomy of the Coumadin Ridge. *Journal of the American Society of Echocardiography: Official Publication of the American Society of Echocardiography*.

[40] Elgendy IY, Conti CR (2013). Caseous calcification of the mitral annulus: a review. *Clinical Cardiology*.

[41] Pradella S, Verna S, Addeo G, Oddo A, Miele V (2019). Caseous Calcification of the Mitral Annulus. *Journal of Radiology Case Reports*.

[42] Hrabak-Paar M, Hübner M, Stern-Padovan R, Lušić M (2011). Hemangioma of the interatrial septum: CT and MRI features. *Cardiovascular and Interventional Radiology*.

