# Supplementary material for: Multimodality Imaging of Cardiac Myxomas
Source: Rev Cardiovasc Med. 2024 Jun 3;25(6):204. doi: 10.31083/j.rcm2506204 (PMC11270062; doi:10.31083/j.rcm2506204)
Supplement: Supplementary file 1 [file 2153-8174-25-6-204-s1.docx]

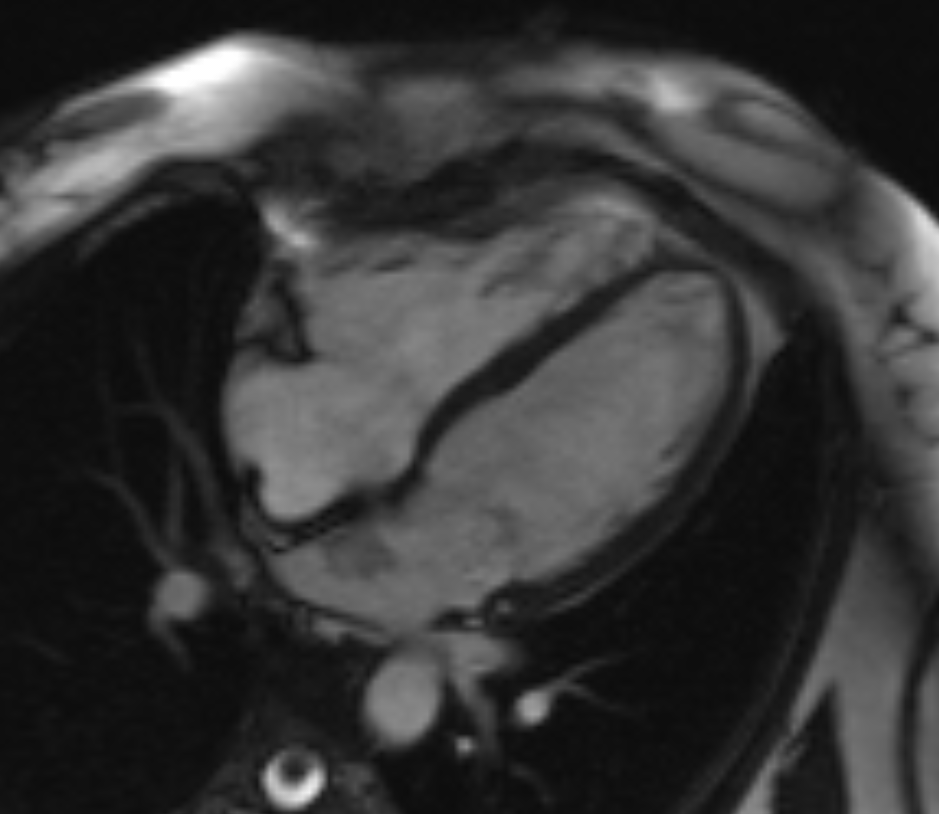


**Supplementary Fig. 1. SSFP cine imaging showing a four-chamber view in a patient with a papillary left atrial myxoma attached to the interatrial septum.** SSFP, steady-state free precession.


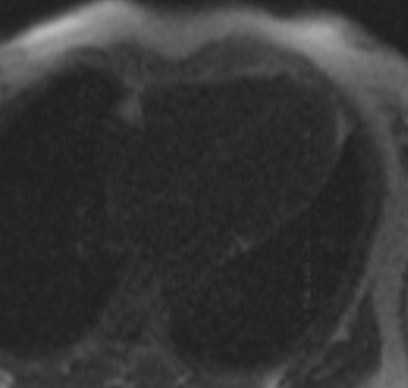


**Supplementary Fig. 2. First-pass perfusion MRI showing a four-chamber view in a patient with left atrial myxoma.**
